# Sociolinguistic Typology and Sign Languages

**DOI:** 10.3389/fpsyg.2018.00200

**Published:** 2018-02-21

**Authors:** Adam Schembri, Jordan Fenlon, Kearsy Cormier, Trevor Johnston

**Affiliations:** ^1^Department of English Language and Applied Linguistics, University of Birmingham, Birmingham, United Kingdom; ^2^Languages and Intercultural Studies, Heriot-Watt University, Edinburgh, United Kingdom; ^3^Deafness Cognition and Language Research Centre, University College London, London, United Kingdom; ^4^Department of Linguistics, Macquarie University, Sydney, NSW, Australia

**Keywords:** sign languages, sociolinguistics, typology, language complexity, morphology, linguistic diversity

## Abstract

This paper examines the possible relationship between proposed social determinants of morphological ‘complexity’ and how this contributes to linguistic diversity, specifically via the typological nature of the sign languages of deaf communities. We sketch how the notion of morphological complexity, as defined by Trudgill (2011), applies to sign languages. Using these criteria, sign languages appear to be languages with low to moderate levels of morphological complexity. This may partly reflect the influence of key social characteristics of communities on the typological nature of languages. Although many deaf communities are relatively small and may involve dense social networks (both social characteristics that Trudgill claimed may lend themselves to morphological ‘complexification’), the picture is complicated by the highly variable nature of the sign language acquisition for most deaf people, and the ongoing contact between native signers, hearing non-native signers, and those deaf individuals who only acquire sign languages in later childhood and early adulthood. These are all factors that may work against the emergence of morphological complexification. The relationship between linguistic typology and these key social factors may lead to a better understanding of the nature of sign language grammar. This perspective stands in contrast to other work where sign languages are sometimes presented as having complex morphology despite being young languages (e.g., Aronoff et al., 2005); in some descriptions, the social determinants of morphological complexity have not received much attention, nor has the notion of complexity itself been specifically explored.

## Introduction

In this paper, we examine the possible relationship between proposed social determinants of morphological complexity (Trudgill, 2011), the typological nature of the sign languages of deaf communities, and how this contributes to an understanding of linguistic diversity. We review the notion of morphological complexity as defined by Trudgill and how it applies to the grammar of sign languages, with a focus on British Sign Language (BSL), Australian Sign Language (Auslan) and American Sign Language (ASL). We then discuss the sociolinguistic situation of sign languages.

## Sociolinguistic Typology

Interest in social structures and linguistic diversity dates back at least a century, as pointed out by Perkins (1992). Based on cross-linguistic evidence, a number of scholars have proposed that spoken languages which undergo extensive second language acquisition by adults appear to have relatively less inflectional complexity (Kusters, 2003; Dahl, 2004; McWhorter, 2007; Wray and Grace, 2007; Miestamo et al., 2008; Sampson et al., 2009). This would suggest that the default state for human languages (i.e., those which lack a history of extensive second language acquisition by adults) is a high degree of morphological complexification, as appears to be true of languages such as the Athabaskan language Navajo (with its highly irregular verbal system) or Yimas (with its rich tense system) spoken in Papua New Guinea. As a result, the moderate degree of morphological complexity of languages like English and French might thus be viewed as a ‘sociohistorical anomaly’ (McWhorter, 2012), resulting from the particular sociolinguistic histories of these two major languages.

Trudgill (2011) introduced the term *sociolinguistic typology:* a ‘sociolinguistically informed’ approach to linguistic typology. This approach assumes that, despite a common set of communicative pressures and cognitive abilities in all humans, different types of languages develop in different places and at different points in time partly as a result of the influence of varying sociolinguistic situations. In particular, this theory proposes that there are specific distinctive social characteristics of speech communities that mold the grammatical organization of their languages. Trudgill (2011) proposed the following factors: (1) population size, (2) social network density, (3) degree of communally shared information, (4) social stability, and (5) degree of language and dialect contact. Morphological complexification, Trudgill suggests, tends to be found in languages used by small communities, composed of dense social networks, with high degrees of communally shared information and social stability, and stable situations of language contact. Stable language contact situations refer here to multilingual communities in which one or more languages are learned as children, as opposed to language contact situations in which large numbers of adults learn a second or additional language, perhaps as the result of some significant social change (e.g., displacement caused by war).

## Morphological Complexity

What does Trudgill (2011) mean by morphological ‘complexification’? He proposes that it consists of the following factors: high degrees of (1) irregularity, (2) morphological opacity, (3) syntagmatic redundancy and (4) morphological marking of categories such as tense, gender, voice etc. Trudgill (2011) illustrates (1) by discussing the irregular system of noun declension in Faroese, with the paradigm for the noun *dagur* ‘day’ showing, for example, completely unrelated forms for accusative [d𝜀a], genitive [daås] and dative case [de:ji] (compare this to the more regular system for *batur* ‘boat,’ with accusative *bat*, genitive *bats* and dative *bati*). By (2), Trudgill (2011) is referring to the notion that the relationship of form and meaning should be as transparent as possible (Kusters, 2003). In a dialect of North Frisian, however, Trudgill reports that, depending on the syntactic context, the infinitive form of ‘do’ has several variant morphological forms (i.e., allomorphs), with it appearing either as *douen*, *doue* or *dou*. Trudgill (2011) illustrates (3) with data from East Flemish dialects in which subject arguments involves triple-marking as in *we zulle-me wij dat doen* ‘we shall do that’ (literally ‘we shall-we we that do’). Lastly, with (4) he explores how the morphological marking in the demonstrative system in some dialects of Norwegian has evolved a three-way distinction between proximal demonstratives *denne/dette/desse* which are equivalent to ‘this’ in English, distal demonstratives *danna/data/dassa* which are similar to English ‘that’ but are used for something that the speaker can point to in contrast to a third type of demonstrative – i.e., the forms *den/dae/dei* which refer to something that is not visible but has been recently mentioned in the conversation.

These aspects of morphological complexity, Trudgill (2011) claims, predominate in smaller, dense, stable communities without large-scale adult second language contact. In fact, many of the examples he describes in Faroese, Frisian, Flemish and Norwegian have emerged in small dialect speaking communities, and represent complexifications in comparison to more standard varieties of each language. He suggests that, as all of these features appear to be difficult for post-critical-period adult learners to master, this reflects that fact that one expects to see morphological simplification – i.e., the reduction in features (1) to (4) – in languages spoken by larger communities with looser social networks that have greater numbers of adult second language learners. Evidence supporting this hypothesis includes a recent study, for example, showing that spoken languages with large numbers of adult second language learners tend to lose nominal case systems (Bentz and Winter, 2013).

## Morphological Complexity and Sign Languages

We would like to focus here on how Trudgill’s (2011) notion of sociolinguistic typology can inform, and can be informed by, the study of sign languages of deaf communities. To our knowledge, this notion has only been partly explored in relation to sign languages (Meir et al., 2012), and the specific predictions of Trudgill’s proposal have not yet been applied to the languages of deaf communities. Sign languages can be divided into two very broad subclasses: (1)‘macro-community’ sign languages which may be used across an entire national deaf community, such as BSL, Auslan, ASL, German Sign Language (DGS) and Taiwan Sign Language (TSL), and (2) ‘micro-community’ sign languages which are used by smaller communities within a nation state, such as the so-called ‘village sign languages’ Kata Kolok in Bali and Al Sayyid Bedouin Sign Language in Israel (see Schembri, 2010 for a description of these two community types). These two types of sign language have developed in quite different social situations, so below we explore how they may provide an interesting test case for the proposal by Trudgill (2011), albeit with some important qualifications.

First, we consider how the notion of morphological complexity might apply to sign languages. Applying Trudgill’s (2011) theory to sign languages is controversial because there is little consensus on how some aspects of their structural organization are best analyzed. Sign languages are often described as morphologically complex languages (e.g., Supalla, 1982, unpublished). Indeed, some researchers have characterized the fact that sign languages appear to have complex morphology despite being young languages a ‘paradox’ (e.g., Aronoff et al., 2005). In contrast, a small number of linguists (e.g., Bergman and Dahl, 1994; Liddell, 2003a) have described sign languages as inflectionless languages, but this view is not widely accepted. After a brief overview of morphology in sign languages, we will work through each of the main features of morphological complexity that Trudgill (2011) discusses, with a focus on BSL, Auslan and ASL (the sign language varieties with which the authors of this paper are most familiar). As we will see, it appears that Trudgill’s notion of morphological complexity and the social determinants associated with it offer some fresh insights into this debate about the structure of sign languages: drawing on this work, we might argue that there is, in fact, no ‘paradox’ to solve.

**FIGURE 1:**
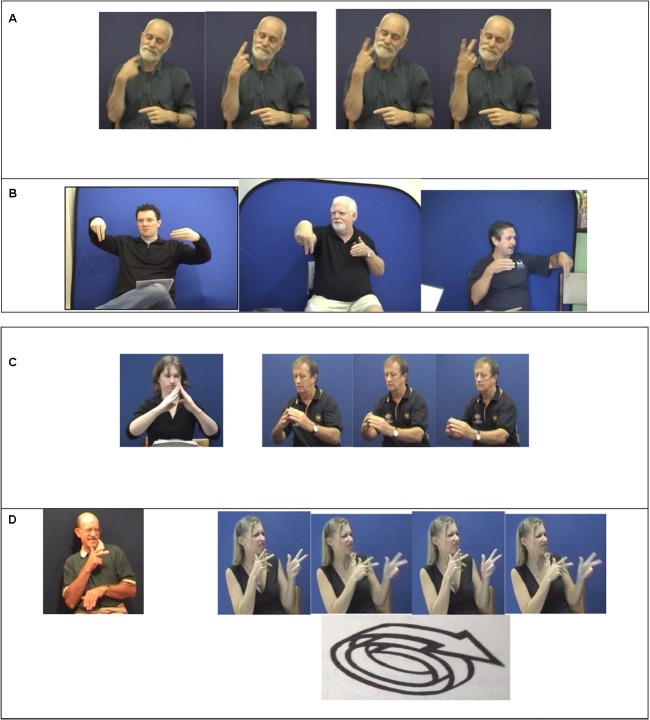
**(A)** TOMORROW and IN-TWO-DAY’S-TIME in Auslan. **(B)** Various Auslan depicting sign constructions. **(C)** Auslan HOUSE versus Auslan HOUSE [PLURAL]. **(D)** Auslan JOKE versus Auslan JOKE [CONTINUATIVE].

First, we provide a little background about sign language structure. Formationally, signs in BSL, Auslan and ASL are composed of contrastive hand configurations, locations on the body or in the space around the signer, movements of the hands, and non-manual features, such as mouth gestures and facial expressions. Morphologically, these formational features may be modified to convey a range of meanings, some of which we explain in more detail below (Sutton-Spence and Woll, 1999; Liddell, 2003a; Johnston and Schembri, 2007). Many of these morphological patterns are widely found in unrelated sign languages, perhaps because they are clearly iconically motivated. For example, time-related signs may incorporate numeral handshapes to show number (e.g., TOMORROW versus IN-TWO-DAY’S-TIME in Auslan in **Figure 1A**). A subset of verb signs, which we will refer to here as *indicating verbs*, may be directed toward locations associated with the referents of the verb’s arguments, as we see in **Figure 3**. Another category of verb signs, known as *classifier constructions* or *depicting signs*, include handshape morphemes that represent classification of a referent into a number of semantic or shape categories. These handshapes combine with movement and spatial components to build complex iconic representations of the specific referent in motion, its relative location and/or its distribution, as we can see in **Figure 1B**. This example shows three possible combinations of a Auslan classifier handshape for person in relation to another classifier handshape for vehicle. These forms represent perhaps the most complex constructions in signs languages, but researchers do not agree on the most appropriate morphological analysis (e.g., Liddell, 2003b). For example, do the changes in relative location in the sign in **Figure 1B** act as discrete morphemes, or are they some kind of gradient gestural representation? In addition to alternations of distinctive formational features of a sign, reduplication of a subset of nouns is used to signal plurality (e.g., Auslan HOUSE versus HOUSE[PLURAL], see **Figure 1C**). Fast or slow reduplication of some verb signs may be used to signal habitual versus continuative aspect (as in Auslan JOKE versus JOKE[continuative] in **Figure 1D**). The rich system for modification of signs is what contributes to the claim by many sign language linguists (e.g., Aronoff et al., 2005) that sign languages are morphologically complex languages.

In terms of Trudgill’s (2011) criteria for morphological complexity, however, the picture seems more mixed, as few of the phenomena identified as morphologically complex by sign linguists (e.g., classifier constructions) fit into his definition. First, none of these three sign languages (BSL, Auslan, or ASL) exhibit high levels of irregularity in any of the morphological phenomena described above. There are a very small number of irregular negative verb and modal forms in each sign language, including CAN and CANNOT in Auslan and in ASL; SHOULD and SHOULD-NOT in BSL, and HAVE and HAVE-NOT in BSL. Some of the negative forms in BSL/Auslan, however, appear to involve a now unproductive negative suffix, as in DISAGREE (cf. AGREE). This suffix appears to be related to the negative lexical item in BSL/Auslan which can mean ‘not have,’ ‘did not,’ ‘without’ etc. There are also irregular forms meaning ‘people’ in Auslan and BSL (unrelated to signs meaning ‘person’). Apart from these small number of examples, however, there are few other examples of irregularity attested (see BSL SignBank and Auslan SignBank for examples of these signs^1^^,^^2^).

**FIGURE 2:**
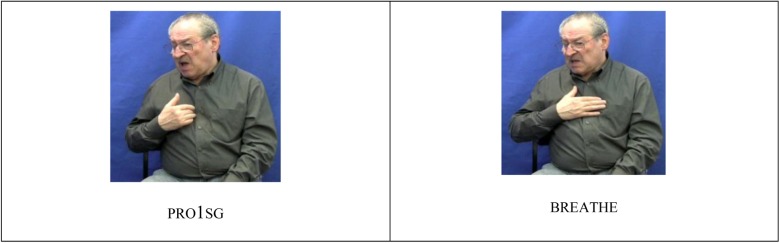
Handshape assimilation in PRO1SG.

There is only limited allomorphy in ASL, BSL and Auslan that cannot be predicted on the basis of morphophonemic processes. For example, in all three sign languages, there is a high degree of variation in the handshape in first person singular pronouns, with the pointing sign directed to the chest appearing as an extended index finger in isolation, but often as some other handshape in connected signing (as we see in **Figure 2** BSL PRO1SG BREATHE ‘I breathe’ where the handshape in the first person pronoun has all fingers extended, matching the handshape of the following sign BREATHE). Empirical studies indicate that this variation may be conditioned in part by the handshape of the following sign (i.e., it is due to co-articulation, see Bayley et al., 2002; Fenlon et al., 2013). Some isolated examples of unpredictable allomorphy do occur in verbs. In one regional variety of Auslan, there are two forms of the non-first person to first person form of the sign GIVE. The form with the Y handshape (i.e., a little finger and thumb extended from the fist), anecdotal reports suggest, cannot be modified for first to non-first person marking^3^. In ASL, there is a non-first person to first person marked form for CONVINCE that is directed toward a location on the neck, unlike other forms of the verb produced in the signing space in front of the signer’s chest. The first person object form has been argued to be an idiosyncratic form (Lillo-Martin and Meier, 2011). However, it could be argued that this form is actually similar to other first person object forms for other indicating verbs which are directed toward particular parts of the body but otherwise are predictable in form (e.g., REMIND, LOOK-AT, etc.).

There is limited syntagmatic redundancy in ASL, BSL, and Auslan, with plural marking of most nouns being optional, for example, even when the nominal occurs with a lexical quantifier or verb modified for number.

ASL, BSL, and Auslan do not employ any morphological markers for gender, tense, or voice. Although some scholars claim that ASL does mark for tense and passive voice (Neidle et al., 1999; Janzen et al., 2001), the claims are based on syntactic, rather than morphological, phenomena. The marking of aspect mentioned above is clearly iconically motivated and does not appear highly grammaticalized in Auslan (Gray, 2013). Furthermore, the aspect marking system is predictable: it involves the reduplication of punctual verbs marking habitual aspect, for example, whereas a similar modification for durative verbs represents durational aspect. In some sign languages, in fact, aspect marking has been considered ideophonic (Bergman and Dahl, 1994).

Genitive case is optionally marked on nouns in Auslan and some varieties of BSL (Johnston and Schembri, 2007; Cormier and Fenlon, 2009): a possessive marker that is based on fingerspelled ‘-s’ (borrowed from English) is sometimes used, as in (1). ASL also has a possessive marker based on a modified form of fingerspelled ‘-s’ which is also optional (Pichler et al., 2008). This appears to be an example of morphological complexification as a result of language contact.

(1) MOTHER POSSESSIVE-S SISTER ‘mother’s sister’

Indicating verbs appear to share some characteristics with person and number agreement in spoken languages (Sandler and Lillo-Martin, 2006; Johnston and Schembri, 2007). This modification has been called ‘agreement’ because it was originally assumed that the form of the verb reflects aspects of the form or semantics of the subject or object noun phrase. In fact, these modifications, like pointing used by non-signers, actually most often reflect the location of a present referent, or the association between an absent referent and a location in the space around the signer’s body (Liddell, 2003a; Fenlon et al., in press). This is arguably quite different from what we see in spoken language agreement systems (Corbett, 2006), and there is considerable debate in the literature about whether it should be called an agreement system at all (e.g., Liddell, 2011; Lillo-Martin and Meier, 2011). Regardless of this debate, it is clear from studies of BSL and Auslan data that this modification is not obligatory (e.g., de Beuzeville et al., 2009; Fenlon et al., in press), as one would expect from a canonical agreement system (Corbett, 2006).

**FIGURE 3:**
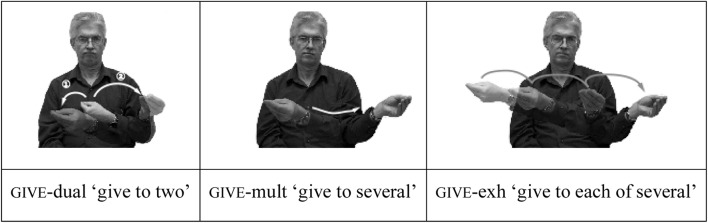
ASL plural forms of indicating verb GIVE.

Indicating verb signs may also be modified for number. An optional alternation of location features and reduplication is used to represent number and distribution of object arguments, as shown in **Figure 3**. With two object arguments, the sign may reduplicate to different locations, or may use a two-handed construction (‘dual inflection’). With more than two, a sweeping movement may be added across the signing space (‘multiple inflection’). Multiple reduplications may signal marking for distribution (the ‘exhaustive inflection’). Again, these modifications are clearly iconically motivated, and do not appear to be obligatory for any sign language.

Overall, it might be argued that BSL, Auslan, and ASL are languages with relatively little obligatory inflection and, based on Trudgill’s (2011) criteria, low to moderate levels of morphological complexity (in contradistinction to Aronoff et al., 2005). Indeed, previous analyses have compared ASL, BSL, and Auslan grammar to spoken language creoles (Fischer, 1978; Ladd and Edwards, 1982; Johnston, 1989). Aronoff et al. (2005) pose this similarity to creoles as a “young language puzzle”: i.e., why is it that sign languages are similar in some ways to spoken language creoles and yet they have complex morphology? Our response is that sign languages, by Trudgill (2011)’s definition, are not as morphologically complex as previously assumed.

## Social Structure and Sign Language Communities

So, what about the social factors at play in deaf communities? Sign language communities tend to be small, but not as small as many spoken languages. For example, Lupyan and Dale (2010) show that the median number of speakers of the 6,192 languages cataloged by Ethnologue is only 7000, although the mean is over 828,000. The total number of signers in North America, the United Kingdom and Australia numbers in the thousands (although this is likely to be in the hundreds of thousands in the North American case), so all of these sign languages would have a lower number than the mean for all languages given in Ethnologue, with only Auslan possibly approaching the much lower median. In terms of the density of social networks, there has been relatively little research into the network densities of macro-community sign languages (the work of Morris, 2016, being the only example). A small number of deaf individuals are from deaf families, work with deaf people and have deaf partners, and this core of the deaf community might have dense social ties with other signers. Over 95% of deaf people, however, are from hearing families (Mitchell and Karchmer, 2004). It is also likely that most deaf adults work with hearing people, and thus they have considerable contact with social networks that do not include people who can sign. It is not clear how to operationalize the variable related to the degree of communally shared information. This is likely to be high in terms of deaf community specific information, but access to information about the wider community is often limited and inconsistent, as the provision of sign language interpreting and captioning on broadcast video is patchy in deaf communities. With regards to social stability, deaf communities are undergoing a period of social change, with traditional centralized schools for deaf children closing, and deaf clubs having increasingly less importance. Both these factors are leading to changing patterns of language transmission. Given only a minority of signers who have ASL, BSL, or Auslan as a first language from signing deaf parents (e.g., Fischer, 1978; Mitchell and Karchmer, 2004), many deaf adults thus acquire these sign languages from other deaf children in primary or secondary school, or in early adulthood in deaf clubs. Some of these deaf adults may not have fully acquired English, and thus may have learnt these sign language varieties as delayed first languages (e.g., Emmorey, 2002). In fact, together with hearing adult second language learners of ASL, Auslan, and BSL, non-native deaf signers constitute the overwhelming majority of the signing community. Together with extensive exposure to spoken and written English, native signers are in constant contact with delayed first language and second language learners. This leads to a sociolinguistic situation that is quite unique, although with some similarities to pidgin language contact situations in which nobody is a native speaker of the variety being used to communicate across language barriers (cf. Fischer, 1978).

## Morphological Complexity in Village Sign Languages

**FIGURE 4:**
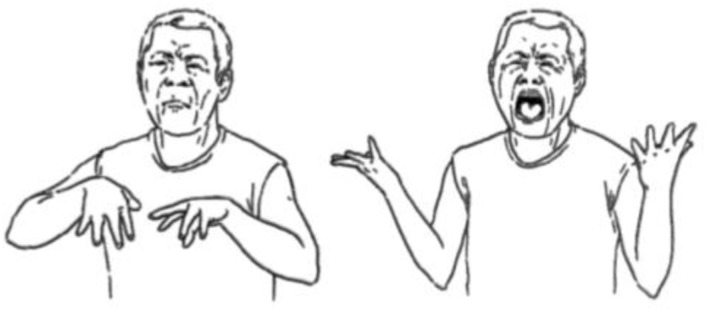
Mouth gesture ‘pah’ in Kata Kolok (from De Vos, 2012) [reproduced with permission].

One might predict that the relatively more dense, stable environments of some micro-community sign languages, such as Kata Kolok, might provide an environment in which complexification is more likely to emerge. We need more research to explore this claim (see Zeshan and De Vos, 2012), but there are some possible hints in the literature. For example, we see some possible complexification in the pronoun and verb systems in Kata Kolok, where the grammar exhibits distinctions in person and aspect marking (Trudgill’s criterion 4, see above). While pointing signs are used for present referents, list buoys (where signers point to fingers on their non-dominant hand, often used to refer to a list of items, cf. Liddell, 2003a) are reportedly used for absent referents (De Vos, 2012). Both pointing signs and list buoys exist in other sign languages, but studies appear to suggest the use of these systems is allocated different grammatical functions categorically in Kata Kolok. Another example might be the emergence of a mouth gesture in Kata Kolok (closed mouth opening, resembling the syllable ‘pah’, see **Figure 4**) which co-occurs with manual verbs to indicate perfective aspect (De Vos, 2012). This is a type of aspect marking which represents an increase in morphological complexity (a similar mouth gesture has been identified in other sign languages, although it does not appear to have the same grammatical role). Perfective aspect marking in ASL, BSL, and Auslan, however, involves the grammaticalization of a manual lexical verb sign meaning ‘finish’ (e.g., Johnston et al., 2015). Therefore, it may be the case that micro-community sign languages provide more dense, stable environments compared to macro-community sign languages, and it is here that we might see some emergent complexification, but more detailed investigation needs to be undertaken.

## Conclusion and Future Directions

In this article, we have briefly explored the idea that socio-cultural and other non-linguistic factors can contribute to linguistic diversity using Trudgill’s (2011) framework of sociolinguistic typology, and we have discussed this proposal with regards to sign languages used by deaf communities for the first time. We have argued that the unique sociolinguistic situation and language transmission patterns of sign languages may contribute as a factor (in addition to the relative youth of sign languages) to explain their relative lack of morphological complexification. This conclusion is controversial since sign languages are sometimes presented as morphologically complex languages that present a puzzle for linguistic theory when their youth is taken into consideration. However, when we apply Trudgill’s notion of linguistic complexity, as we have done here, a clearer picture of the nature of sign languages and their relationship to their sociolinguistic situation emerges. If Trudgill is correct, even considerably longer histories may not lead to morphological complexification in macro-community sign languages. In future, more research needs to be carried out on the specific sociolinguistic situation of sign languages, particularly with regards to the relative impact of social network density on these languages, as well as their youth and propensity for highly iconic structures (e.g., Cuxac and Sallandre, 2007).

## Author Contributions

AS, JF, KC, and TJ all made substantial contributions to the conception of the work and the interpretation of data. AS led on the writing of the paper, with JF, KC, and TJ all contributing to revising it critically for intellectual content and style. AS, JF, KC, and TJ all gave final approval of the version to be published and agreed to be accountable for all aspects of the work in ensuring that questions related to the accuracy or integrity of any part of the work are appropriately investigated and resolved.

## Conflict of Interest Statement

The authors declare that the research was conducted in the absence of any commercial or financial relationships that could be construed as a potential conflict of interest.

## Footnotes

1 http://bslsignbank.ucl.ac.uk

2 http://www.auslan.org.au

3 http://www.auslan.org.au/dictionary/words/give%20back-1.html
